# Case report: Primary familial brain calcification associated with a rare *PDGFRB* variant, coexisting with nontraumatic osteonecrosis of the femoral head

**DOI:** 10.3389/fnins.2024.1381840

**Published:** 2024-05-27

**Authors:** Conghui Cao, Jing Luo, Xiaoli Wang

**Affiliations:** ^1^Department of Endocrinology and Metabolism, Institute of Endocrinology, NHC Key Laboratory of Diagnosis and Treatment of Thyroid Diseases, The First Hospital of China Medical University, Shenyang, China; ^2^Department of Endocrinology and Metabolism, Tieling Central Hospital, Tieling, China

**Keywords:** primary familial brain calcification, *PDGFRB*, osteonecrosis of the femoral head, case report, whole-exome sequencing

## Abstract

Primary familial brain calcification (PFBC) is a rare genetic neurodegenerative disorder characterized by bilateral calcifications in the brain. PFBC may manifest with a broad spectrum of motor, cognitive, and neuropsychiatric symptoms. Several causal genes have been identified in PFBC, which are inherited as both autosomal dominant and autosomal recessive traits. Herein, we present the case of a Chinese family diagnosed with PFBC. The family members carry a rare heterozygous variant (p. R334Q) in exon 7 of *platelet-derived growth factor receptor β* (*PDGFRB*) gene. The platelet-derived growth factor-B/PDGF receptor *β* (PDGF-B/PDGFRβ) signaling pathway plays a crucial role in pericyte development in various organs and tissues. Notably, this variant uniquely coexists with nontraumatic osteonecrosis of the femoral head. Additionally, we reviewed previous studies on PFBC-causing variants in *PDGFRB*.

## 1 Introduction

Primary familial brain calcification (PFBC), also known as idiopathic basal ganglia calcification, or Fahr disease, is a rare genetic neurodegenerative disorder. It is characterized by bilateral calcifications in the brain, primarily in the basal ganglia, and can also affect other brain regions, such as subcortical white matter, cerebellum, and thalamus (Tadic et al., 2015; Westenberger et al., 2019).

PFBC may manifest with a spectrum of nonspecific neuropsychiatric symptoms, including movement disorders, cognitive impairment, and psychiatric manifestations (Tadic et al., 2015; Donzuso et al., 2019; Westenberger et al., 2019). However, approximately one-third of patients with PFBC remain clinically asymptomatic throughout their lives (Chen et al., 2023).

Several variations in certain genes have been identified as causative factors for PFBC, including variations in the genes *SLC20A2*, *PDGFRB*, *PDGFB*, and *XPR1* for autosomal dominant PFBC and those in the genes *MYORG*, *JAM2*, and *CMPK2* for autosomal recessive PFBC (Keller et al., 2013; Legati et al., 2015; Yao et al., 2018; Westenberger et al., 2019; Schottlaender et al., 2020; Zhao et al., 2022). However, these genetic variations only account for 50–60% of all PFBC cases (Westenberger et al., 2019; Chen et al., 2023).

The PDGF-B/PDGFRβ signaling pathway plays a crucial role in pericyte development in various organs and tissues, including blood vessels, kidneys, and the central nervous system (Andrae et al., 2008). Loss-of-function variants in *PDGFRB* and *PDGFB* are implicated in PFBC (Sanchez-Contreras et al., 2014; Lenglez et al., 2022), contributing to 5 and 12% of genetically confirmed PFBC cases, respectively (Balck et al., 2021; Chen et al., 2023).

Herein, we report the case of a family with PFBC caused by a rare mutation in the *PDGFRB* gene. They also exhibited clinical manifestations of early-onset nontraumatic osteonecrosis of the femoral head (ONFH).

## 2 Case description

**Figure 1:**
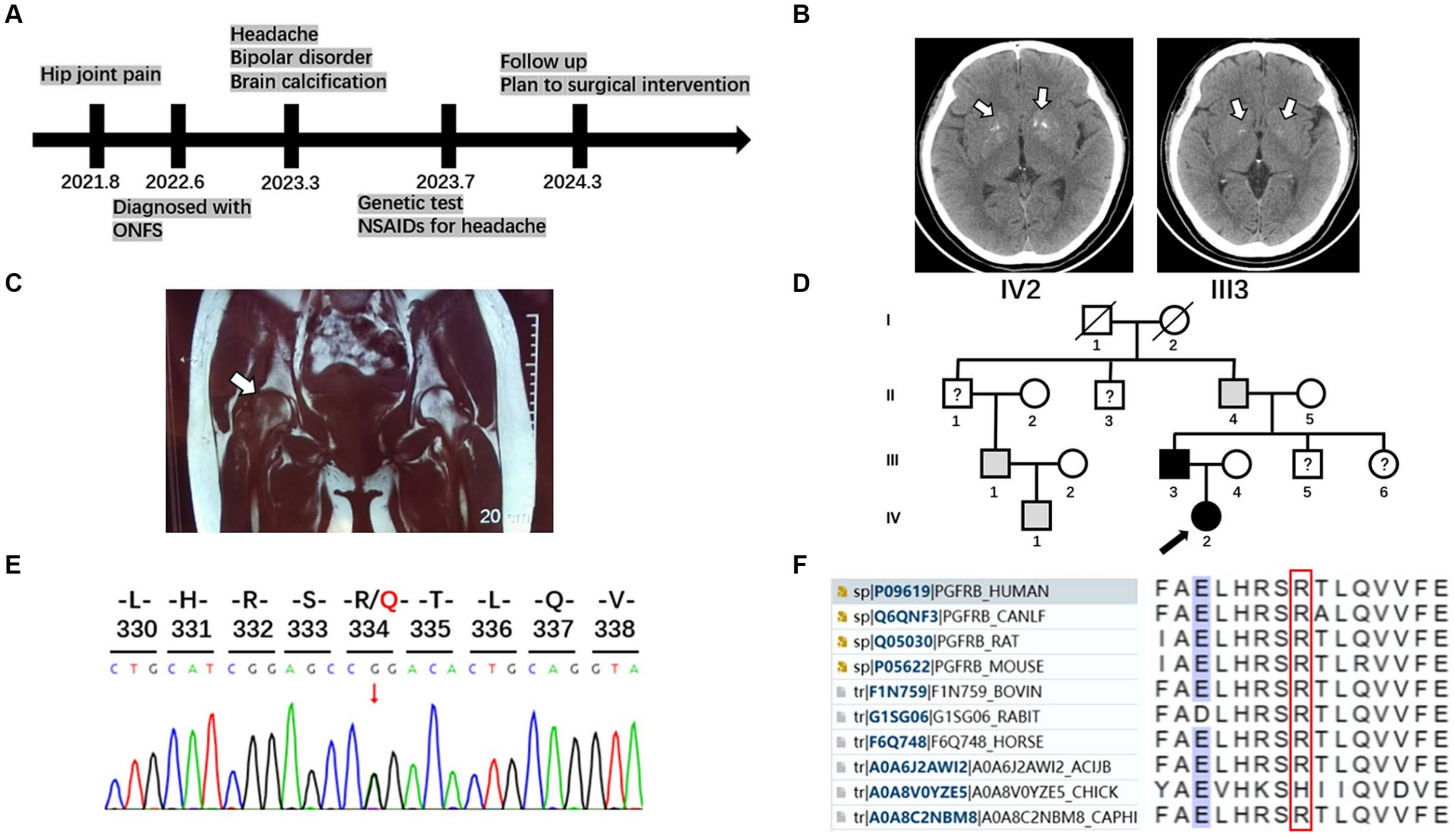
Timeline, brain imaging, MRI of femoral head, pedigrees, and sequencing results of the family with PFBC and ONFH. **(A)** A timeline of historical and current information of the care. **(B)** Brain imaging of the patient and her father. White arrows indicate calcification regions in the bilateral basal ganglia. **(C)** Osteonecrosis of the femoral head. Coronal T1-weighted magnetic resonance imaging displayed slightly hypointense lesions in both femoral heads, with a more pronounced pattern on the right side (white arrow). **(D)** Pedigrees of the family with PFBC with *PDGFRB* mutation R334Q. A black arrow marks the proband. Males and females are indicated by squares and circles, respectively. Symbols with slashes indicate deceased individuals. Black symbols indicate individuals with PFBC and confirmed *PDGFRB* R334Q mutation; gray symbols indicate individuals with similar clinical manifestations without genetic analysis; symbols with question marks indicate individuals with unknown status. **(E)** Sequencing chromatogram of the mutation R334Q in *PDGFRB*. **(F)** Multiple sequence alignment in the *PDGFRB* gene. Amino acids surrounding the mutation position (marked with a red box) are listed.

A 24-year-old woman presented to our neurology department with complaints of occasional headaches and bipolar disorder. Two years ago, she was diagnosed with ONFH during a visit to the orthopedics department due to hip joint pain. The pain progressively worsened, impairing her ability to walk, necessitating the use of crutches and other assistive devices. She has no significant medical or medication history. While occasionally taking nonsteroidal anti-inflammatory drugs (NSAIDs) for headaches, she did not receive specific treatment for other psychoneurological symptoms and ONFH beyond weight-bearing reduction. Currently, she seeks further evaluation for surgical intervention in the orthopedics department (Figure 1A).

Her laboratory tests revealed normal levels of calcium, phosphorus, and parathyroid and thyroid-stimulating hormones in the blood. A head computed tomography (CT) scan showed bilateral basal ganglia calcification (Figure 1B). Coronal T1-weighted magnetic resonance imaging displayed slightly hypointense lesions in both femoral heads, with a more pronounced pattern on the right side (Figure 1C).

Her 50-year-old father had a history of occasional headaches, depression, and ONFH in his youth. He underwent surgical treatment for ONFH but did not receive specialized neurological treatment. His head CT revealed mild bilateral basal ganglia calcification (Figure 1B).

Although the patient’s mother is healthy, her grandfather, uncle, and cousin have not undergone head CT or genetic testing for similar medical histories (Figure 1D).

## 3 Genetic testing

**Figure 2:**
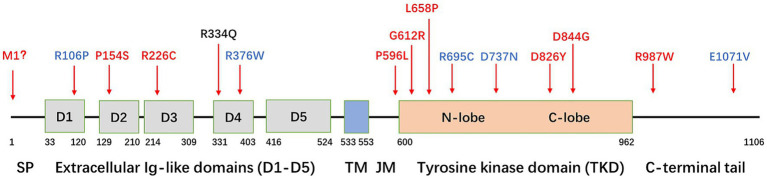
Schematic diagram of the *PDGFRB* gene and localization of variants. SP, signal peptide; TM, transmembrane domain; JM, juxtamembrane domain. Variants in red indicate a confirmed detrimental effect to induce PFBC, variants in blue indicate an unclear relationship with PFBC, and the variant in black indicates the mutation (R334Q) found in this family.

After obtaining written informed consent from the patient’s family, blood samples were collected from the patient and his parents. Genomic DNA was extracted using a blood extraction kit (Tian Jing Biochemical Technology Beijing, Ltd.). Genetic testing was performed using whole-exome high-throughput sequencing technology on the Illumina platform, and the data were analyzed using the Verita Trekker^®^ Variant Site Detection System and Enliven^®^ Variant Site Annotation Interpretation System developed by Berry Genetics (based on the recent version of the dbSNP, 1,000 Genome, gnomAD, CADD, ClinVar, and HGMD database). As a result, a heterozygous variant c.1001G > A (p.R334Q) in the gene *PDGFRB* (NM_002609.4) was discovered in the patient and her father. These variants were confirmed by Sanger sequencing (Figure 1E). No variants were found in other known PFBC-causal or primary nontraumatic ONFH-causal genes. The p.R334Q variant is located in exon 7 of *PDGFRB*, which is within an extracellular immunoglobulin (Ig) – like domain (D4) conserved in mammalian species (Figures 1F, 2). This missense variant was reported in genomAD^1^ at an extreme frequency of 0.00007378. It is predicted to be likely pathogenic according to the ACMG-AMP recommendations (PM1, PM2, PP1, PP3). However, the predicted score by theoretical modelling in the AlphaMissense database^2^ is 0.071, which is likely benign.

## 4 Discussion

PDGFRβ is a cell surface receptor responsible for tyrosine kinase activity expressed in various cells, such as neurons, plexus choroideus, vascular smooth muscle cells, and pericytes. The exact mechanism by which decreased PDGF-B/PDGFRβ signaling leads to intracranial calcification is not fully understood. Furthermore, it is unclear why the brain vasculature is the primary target for the calcification process. This process may be related to enhancing the integrity of the blood–brain barrier and influencing the PDGF Pit-1 pathway (Nicolas et al., 2013; Arts et al., 2015; Vanlandewijck et al., 2015).

PDGFRβ is a protein with five extracellular immunoglobulin-like loops and an intracellular tyrosine kinase domain (TKD). Only a few *PDGFRB* mutations causing PFBC have been reported in the literature (Figure 2). These mutations can be classified into four types.

The first type of mutation is located in extracellular Ig-like domains (D1–D5), causing the receptor to lose its ability to bind to PDGF-BB, such as R106P (D1), P154S (D2), R226C (D3) (Sun et al., 2021; Lenglez et al., 2022), and R334Q (D4). The R334 is highly evolutionarily conserved in mammalian species.

The second type of mutation is located in the TKD, resulting in a loss of tyrosine kinase enzymatic activity. Mutations in this category include P596L (JM), G612R, L658P, D826Y, and D844G (Arts et al., 2015; Ramos et al., 2018; Lenglez et al., 2022). Nearly 50% of the reported mutations (7/15) are located in the TKD.

The third type of mutation results in a decrease in cell surface expression, with M1 being an example. Additionally, mutations in the Ig-like domains D1–D4 may lead to a partial decrease in expression (Wang et al., 2017; Lenglez et al., 2022).

The fourth type of mutation is located in the C-terminal domain, with the most representative being mutation R987W. This mutation affects receptor trafficking, leading to increased internalization and degradation (Arts et al., 2015; Vanlandewijck et al., 2015; Lenglez et al., 2022). Although several other mutations are found in patients with PFBC, functional tests suggest that their impact on *PDGFRB* functions is minimal. Examples of these mutations include R695C, R376W, D737N, and E1071V (Sanchez-Contreras et al., 2014; Lenglez et al., 2022). Whether these mutations are sufficient to explain the PFBC phenotype remains unclear.

PFBC presents with three main categories of symptoms: cognitive impairment, psychiatric signs, and movement disorders. However, some individuals may exhibit imaging manifestations without evident symptoms. In the present case, the proband exhibited no other typical symptoms except headaches and emotional disorders. Interestingly, both the patient and her father have early-onset nontraumatic ONFH.

The etiology of nontraumatic ONFH is complex, multifactorial, and not entirely understood. Various factors, such as glucocorticoid use, alcohol abuse, and certain diseases (including hemoglobinopathies, coagulopathies, malignancies, autoimmune diseases, metabolic disorders, and renal failure) can potentially induce ONFH (Rezus et al., 2021). Moreover, some genetic factors may contribute to primary nontraumatic ONFH, possibly linked to hereditary thrombophilia or hybridization. These genetic factors include conditions such as the factor V Leiden mutation, the prothrombin gene G20210A mutation, antithrombin III deficiency, protein C and protein S deficiency, and polymorphisms in *MTHFR* and *PAI-1* (Ali et al., 2014). However, it is noteworthy that high-throughput sequencing did not reveal any variations related to hereditary thrombophilia or hybridization within this family.

It is known that decreased PDGF-B/PDGFRβ signaling can result in pericyte hypoplasia, endothelial hyperplasia, increased vessel diameter, vascularity, and vessel instability (Sanchez-Contreras et al., 2014). However, there is no information on whether these effects on angiogenesis establish an association between PDGF-B/PDGFRβ signaling and ONFH. Thus, we cannot yet ascertain whether the coexistence of PFBC and ONFH in this family is a coincidence or potentially correlated.

Currently, there is no specific therapy for PFBC to reduce brain calcification or prevent its progression. However, in a previous study, patients with PFBC receiving biphasic alendronate treatment showed good tolerance and evidence of overall improvement and stability (Oliveira and Oliveira, 2016). Nevertheless, further studies with a larger sample size and randomized controlled studies are required to confirm the results. Additionally, due to disrupted Pi homeostasis, the potential use of sediment-dependent phase cotransporters for PFBC has been explored (Inden et al., 2022).

## Data availability statement

The datasets presented in this study can be found in online repositories. The names of the repository/repositories and accession number(s) can be found at: https://figshare.com/articles/journal_contribution/Sequencing_results/25139981, 25139981.

## Ethics statement

The studies involving humans were approved by the hospital ethics committee of China Medical University. The studies were conducted in accordance with the local legislation and institutional requirements. Written informed consent for participation in this study was provided by the participants’ legal guardians/next of kin. Written informed consent was obtained from the individual(s) for the publication of any potentially identifiable images or data included in this article.

## Author contributions

CC: Writing – original draft. JL: Writing – original draft. XW: Formal analysis, Methodology, Writing – original draft, Writing – review & editing.

## Footnotes

^1^ https://gnomad.broadinstitute.org/

^2^ https://alphamissense.hegelab.org/
